# The effect of 2D and 3D cell cultures on treatment response, EMT profile and stem cell features in head and neck cancer

**DOI:** 10.1186/s12935-019-0733-1

**Published:** 2019-01-14

**Authors:** Styliani Melissaridou, Emilia Wiechec, Mustafa Magan, Mayur Vilas Jain, Man Ki Chung, Lovisa Farnebo, Karin Roberg

**Affiliations:** 1 Division of Cell Biology, Department of Clinical and Experimental Medicine, Linköping University, Linköping, Sweden; 2 Department of Otorhinolaryngology in Linköping, Anaesthetics, Operations and Specialty Surgery Center, Region Östergötland, Linköping, Sweden; 3 Department of Molecular Medicine and Gene Therapy, Lund Stem Cell Center, Lund University, Lund, Sweden; 4 Department of Otorhinolaryngology-Head & Neck Surgery, Sungkyunkwan University School of Medicine, Samsung Medical Center, Seoul, South Korea

**Keywords:** Spheroids, Cancer stem cells, Epithelial–mesenchymal transition, Head and Neck Squamous Cell Carcinoma, Drug response

## Abstract

**Background:**

Head and Neck Squamous Cell Carcinoma (HNSCC) tumors are often resistant to therapies. Therefore searching for predictive markers and new targets for treatment in clinically relevant in vitro tumor models is essential. Five HNSCC-derived cell lines were used to assess the effect of 3D culturing compared to 2D monolayers in terms of cell proliferation, response to anti-cancer therapy as well as expression of EMT and CSC genes.

**Methods:**

The viability and proliferation capacity of HNSCC cells as well as induction of apoptosis in tumor spheroids cells after treatment was assessed by MTT assay, crystal violet- and TUNEL assay respectively. Expression of EMT and CSC markers was analyzed on mRNA (RT-qPCR) and protein (Western blot) level.

**Results:**

We showed that HNSCC cells from different tumors formed spheroids that differed in size and density in regard to EMT-associated protein expression and culturing time. In all spheroids, an up regulation of CDH1, NANOG and SOX2 was observed in comparison to 2D but changes in the expression of EGFR and EMT markers varied among the cell lines. Moreover, most HNSCC cells grown in 3D showed decreased sensitivity to cisplatin and cetuximab (anti-EGFR) treatment.

**Conclusions:**

Taken together, our study points at notable differences between these two cellular systems in terms of EMT-associated gene expression profile and drug response. As the 3D cell cultures imitate the in vivo behaviour of neoplastic cells within the tumor, our study suggest that 3D culture model is superior to 2D monolayers in the search for new therapeutic targets.

**Electronic supplementary material:**

The online version of this article (10.1186/s12935-019-0733-1) contains supplementary material, which is available to authorized users.

## Background

Head and Neck Squamous Cell Carcinoma (HNSCC) is the sixth most common cancer worldwide with over half a million new cases annually. The 5-year relative survival rate differs greatly and depends on the tumor location and the advancement of the disease [1, 2]. The prognosis is dependent on invasion and local recurrence and the interaction between tumor cells and the surrounding extra cellular matrix with cancer-associated fibroblast facilitating invasion and leading to treatment resistance.

The research initiated by Sutherland et al. has shown that three-dimensional (3D) multicellular spheroids of cancer cells resemble in many aspects solid tumors and serve as a promising model to mimic the in vivo situation [3]. Considerable differences in metabolism and tolerance of hypoxic conditions within the spheroids as well as drug penetration heterogeneity are associated with increased resistance to chemo- and radiotherapy in comparison to two-dimensional (2D) cell cultures [4, 5]. Moreover, the use of different cell populations in tumor spheroids such as fibroblasts or endothelial cells allows obtaining structures that imitate the in vivo tumor also in terms of cellular heterogeneity [4]. The growing research interest in 3D models of different tumor types is focused on assessing the effects of chemo- and/or radiotherapy, studying tumor metastasis, angiogenesis and cancer cell differentiation, apoptosis as well as epithelial–mesenchymal transition (EMT) and cancer stem cells (CSCs) biology [6, 7].

A small subpopulation of CSCs is present in many solid tumors including HNSCC and is well described to participate in tumour invasion and metastasis [8–10]. The theory of cancer stem cells is now widely accepted, and was first shown in breast cancer cells [11]. CSCs are recognized based on cell surface phenotype, including high expression of the CD44 surface marker [12, 13].

Recently two distinct phenotypes of CD44^high^ CSC, namely non-EMT CSC (CD44^high^ ESA^high^) and EMT-dependent CSC (CD44^high^ ESA^low^) have been identified in squamous cell carcinoma [14]. Up to date studies have pointed at a link between the EMT and CSC phenotype. EMT plays an important role in the acquiring and maintenance of stem cell-like properties and is associated with elevated expression of mesenchymal markers such as Vimentin, N-cadherin, Fibronectin, Snail, Foxc2 and Twist. The enrichment of tumor spheroids in CSCs that are triggered by EMT certainly has implications in the response to anti-cancer treatment [15].

The aim of this study was to generate reliable in vitro 3-D models with properties as close as possible to the in vivo tumor. Therefore, we here use HNSCC cell lines in low passages and compare phenotypic differences as well as differences in treatment response rate between 2D and 3D cultures.

## Materials and methods

### Cell lines and culture conditions

Five HNSCC cell lines, namely LK0858B, LK0902, LK0917, LK1108 and LK1122 from the Linköping University collection were used in this study (Table 1). All cell lines were derived from tissue specimens from patients diagnosed with HNSCC and were cultured in Keratinocyte-SFM supplemented with antibiotics (penicillin 50 U/ml, streptomycin 50 µg/ml), and 10% FBS (all from GIBCO). The cells were given fresh culture media twice per week and were subcultured at confluence after detaching the cells with 0.25% trypsin + 0.02% EDTA at a weekly split ratio of approximately 1:2 or 1:3. Cultures in passages 10 to 25 were used in all experiments. Cells were screened periodically for mycoplasma contamination using DAPI staining and/or the Universal Mycoplasma Detection Kit (ATCC, USA).

**Table 1:** Origin and tumor characteristics of the investigated cell lines

Cell line	Primary tumor location	TNM	Gender
LK0858B	Tongue	T3N0M0	F
LK0902	Tongue	T1N0M0	F
LK0917	Gingiva	T4N1M1	M
LK1108	Hypopharynx	T2N0M0	F
LK1122	Larynx	T3N1M0	M

TNM classification according to the International Union against Cancer (UICC, 2002)

### Spheroid preparation

Tumour spheroids were generated by seeding 6000 to 8000 cells/well in ultra-low attachment (ULA) 96-well round-bottomed plates (Corning, USA) and cultured for 1 day, 3 days and 7 days at standard culture conditions.

### Sample preparation and immunohistochemistry (IHC)

Spheroids were fixed in 4% paraformaldehyde (Santa Cruz Biotechnology, USA) over night at 4 °C, thereafter rinsed with PBS, stained with 0.1% toluidine blue D (Merck, USA) and then centrifuged down in 2% agarose. The agarose blocks containing spheroids were dehydrated using an accelerating ethanol series followed embedding in paraffin wax (Merck, USA). Sections of 4 µm thickness were mounted on Super Frost Plus slides (Thermo Fisher Scientific, USA), dried overnight and then incubated at 60 °C for 1 h.

Standard hematoxylin and eosin stained slides of the spheroids were first evaluated to confirm spheroids quality and content in paraffin blocks. New sections from the spheroids were thereafter mounted on positively charged slides and deparaffinized in Aqua dePar (Biocare Medical, USA). Sections were pre-treated with 10 mM citrate buffer retrieval solution (DakoCytomation, Denmark) in a hot water bath (up to 100 °C) for 40 min, blocked with Envision peroxidase block (BCPX968) for 5 min, and incubated for 30 min at room temperature with an antibody against Ki-67 (M7240, Dako, Sweden). Sections were thereafter stained with the EnVision System-HRP (DAB) kit (DakoCytomation, Denmark), followed by counterstaining for 1 min with Tacha’s hematoxylin.

### Immunofluorescence

Spheroids were fixed and embedded as described above. For immunofluorescence (IF) staining, 4 μm sections were cut from the paraffin embedded spheroid and permeabilized with 0.2% triton X100 and 0.5% BSA-PBS for 15 min. Sections were then exposed to blocking buffer (0.5% BSA-PBS) for 20 min. Next primary anti-pan Cytokeratin antibody [AE1/AE3] (Abcam, USA, ab80826, 1:50) were incubated overnight at 4 °C. After washing, cells were incubated with Goat anti-mouse Alexa Fluor 594 (IgG H&L; Abcam, USA, ab150116, 1:200) secondary antibody for 45 min. Finally, sections were mounted using Vectashield with DAPI (Vector laboratory, USA, H-1500). The staining images were acquired with a confocal microscope (Zeiss, LSM 700).

### TUNEL assay

Cell death was evaluated with the DeadEnd™ Fluorometric TUNEL System (G3250, Promega, USA). Briefly, paraffin section slides were deparaffinized, rehydrated, digested at room temperature and washed in PBS. Sections were then incubated with equilibration buffer for 2 min and immediately incubated with the recombinant terminal deoxynucleotidyl transferase reaction mixture for 60 min at 37 °C. Fluorescein isothiocyanate (FITC)-labeled anti-digoxigenin conjugate was applied to the sections. The reaction was terminated by 2X SSC solution.

#### Assessment of viability and proliferation capacity after cisplatin or cetuximab treatment

Tumor cells were seeded to 96-well ULA plates and cultured for 48 h before drug exposure. Cetuximab (60, 90 and 120 nM; Erbitux^®^, Merck KGaA, Darmstadt, Germany) or cisplatin (1, 2 and 4 µg/ml; Cisplatin Meda, Sandoz A/S, Denmark) was added to selected spheroids or monolayer cells and the cytostatic/cytotoxic effect was determined after another 7 days. Eight spheroids were sampled in an Eppendorf test tube, centrifuged (1000 rpm, 5 min), washed in PBS, centrifuged and then incubated with 200 µl trypsin for 5 to 20 min at 37 °C. Thereafter 200 µl culture media was added, flushed 30 times with a micropipette and subjected for the in vitro clonogenic assay (proliferation) or MTS assay (viability).

Briefly, for the clonogenic assay dissociated spheroid-derived cells were seeded into a 12 well plate and cultured at standard culture conditions for 7 days. After fixation in 4% paraformaldehyde (Santa Cruz Biotechnology, USA), cells were stained with crystal violet (0.04% in 1% ethanol) solution for 20 min at room temperature, washed and air-dried. After solubilization in 1% SDS the clonogenic growth was measured at 550 nm using a Victor plate reader (EG & G Wallac, Sweden).

The viability of treated tumor cells was measured using the CellTiter 96^®^ AQ_ueous_ One Solution Cell Proliferation Assay (Promega, USA). Briefly, treated and untreated tumor cells grown in 2D and 3D (dissociated spheroid-derived cells) were added to a 96 well plate and 20 µl/100 µl medium of the MTS substrate was then added followed by a 3 h incubation in 37 °C. The absorbance of each well was measured at λ = 490 nm with a VersaMax (Molecular Devices, USA) microplate reader. All analyses were performed three times, and the mean values were used for further calculations.

### RT-qPCR

The RT-qPCR analysis was performed on a 7500 Fast Real-Time PCR system (Applied Biosystems, USA). Total RNA was extracted from the cells using the RNeasy Mini Kit (Qiagen, Germany), cDNA was synthesized using the High Capacity RNA-to-cDNA Kit (Applied Biosystems, USA), and TaqMan FAM/MGB probes (Applied Biosystems, USA) were used for the PCR reaction. Amplification of both glyceraldehyde 3-phosphate dehydrogenase (GAPDH) and β-actin was used as an internal standard. The comparative Ct method was applied to determine the fold-difference in expression levels relative to a control sample [16].

### Western blot

Aliquots of protein (30 µg) were subjected to Western blotting. The membranes were incubated with a rabbit polyclonal EGFR antibody (Santa Cruz Biotechnology, USA), a mouse anti-vimentin (Santa Cruz Biotechnology, USA), a mouse anti-NANOG (Santa Cruz Biotechnology, USA), a mouse anti-Sox2 (R&D Systems, USA), a rabbit anti-N-cadherin (Abcam, USA), a rabbit anti-fibronectin 1 (Merck, USA), a mouse anti-CD44 (Cell Signaling, USA) and anti-E-cadherin (Santa Cruz Biotechnology, USA). After washing with TBS-Tween (Merck, USA), the membranes were incubated with HRP-conjugated goat anti-rabbit antibody (Santa Cruz Biotechnology, USA) or HRP-conjugated goat-anti-mouse antibody (Santa Cruz Biotechnology, USA). Equal loading was verified by reprobing the membranes with HRP-conjugated anti-GAPDH antibody (Novus Biologicals, USA). The bands were visualized with Western Blotting Luminol Reagent (Bio Rad, USA) using Chemi Doc™ MP Imaging System (Bio Rad, USA).

### Statistics

Data are presented as the mean ± SD. All experiments were repeated at least three times with duplicates. The data was analyzed using one-way ANOVA followed by Student’s t-test with Bonferroni adjustment using GraphPad Prism software (GraphPad Prism 7, USA). p-values ≤ 0.05 were considered significant.

## Results

### Morphology of HNSCC tumor spheroids

**Fig. 1:**
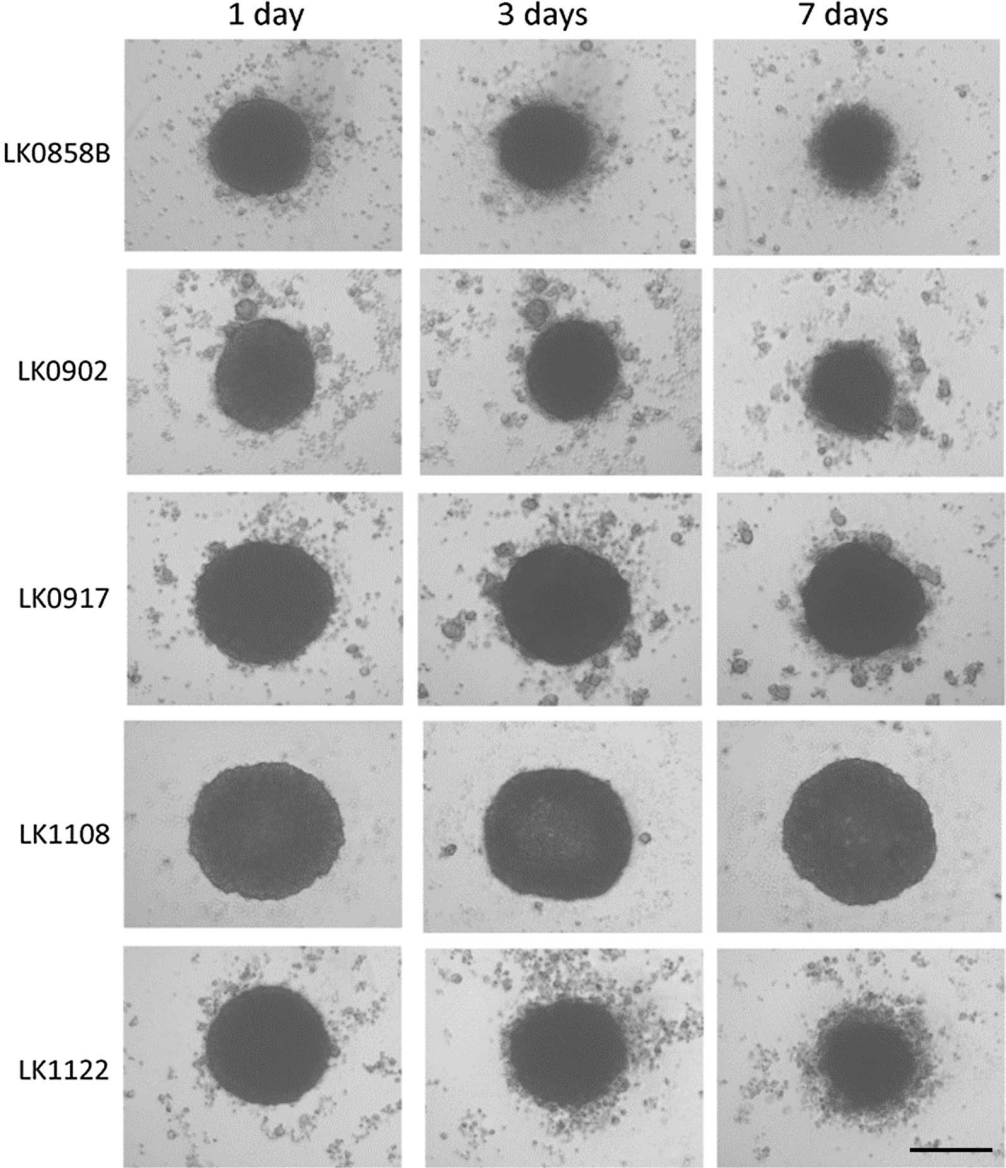
Morphology of HNSCC-derived tumor spheroids. Morphology of HNSCC cell lines cultured as 3D tumor spheroids in ULA 96-well plates. The images were captured by a phase-contrast microscopy at day 1, 3 and 7 in culture; scale bar = 200 µm

**Fig. 2:**
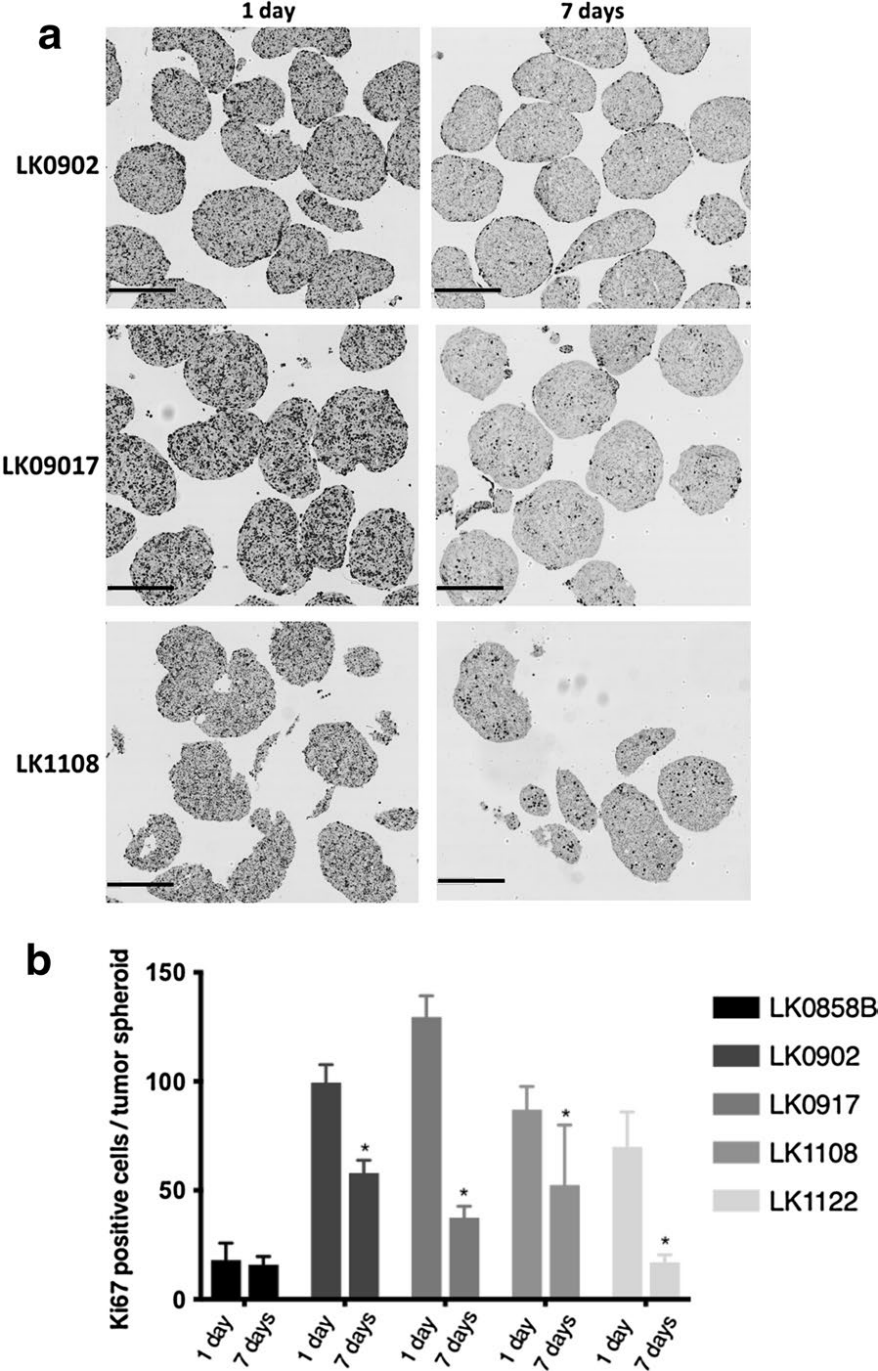
Histological evaluation of HNSCC-derived tumor spheroids. **a** Immunohistochemical staining of HNSCC tumor spheroids with the proliferation marker Ki67 cultured for 1 and 7 days; scale bar = 300 µm. **b** Quantification of Ki67-positive cells in 1 day and 7 days old tumor spheroids; the data are depicted as mean of ± SD, n = 10. *p < 0.05 according to Student’s test

Cells (6000–8000) of each cell line (Table 1) were seeded into 96-well ultra-low attachment (96UL) plates. Figure 1 presents spheroids from five HNSCC cell lines cultured for 1, 3 and 7 days at standard culture conditions. Some morphology differences among the cell lines were observed already after 1 day of culture and was more pronounced after 7 days. In three of the cell lines migration from the spheroids to the medium was observed, which was most clear in the LK1122 spheroids. Additionally, the expression of the proliferation marker Ki67 was assessed at each time point by using immunohistochemistry. A decrease in Ki67 expression was seen in all spheroids after 3 and 7 days of cultivation and after 7 days Ki67 positive cells were mainly found in the outer layer (Fig. 2a). In four out of five cell lines, a significant decrease in Ki67 positive cells was observed after 7 days (Fig. 2b).

Furthermore, the expression of cytokeratin in the tumor spheroids was examined after 7 days with immunofluorescence staining showing a high amount of cytokeratin positive cells (Additional file 1: Figure S1). Moreover, cell death was analyzed with a TUNEL assay but only a low number of apoptotic cells was found in the analyzed tumor spheroids (Additional file 1: Figure S1).

### Expression of CSC-associated markers is more evident than expression of EMT markers in HNSCC-derived spheroids

**Fig. 3:**
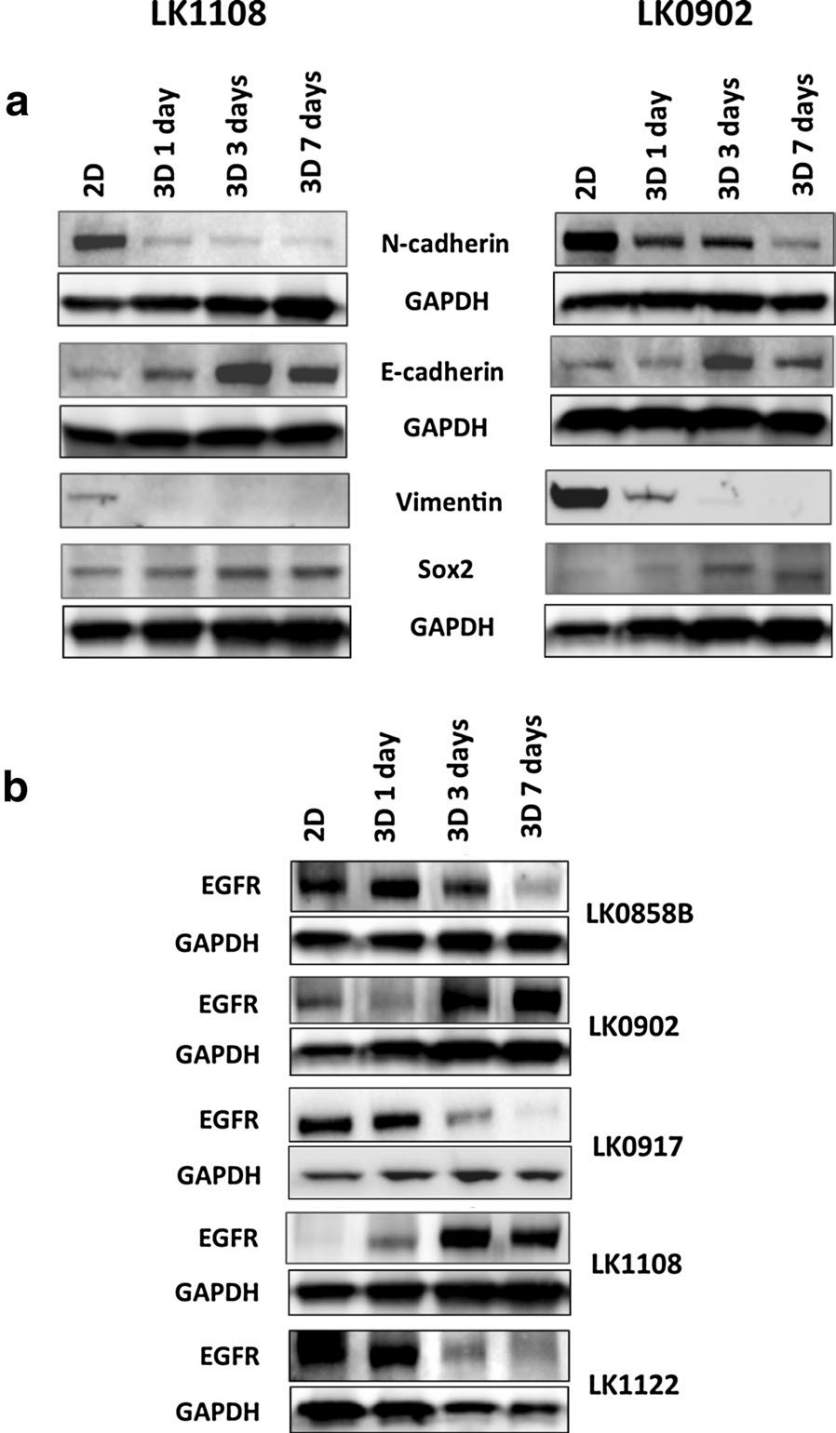
Western blot analysis for HNSCC-derived tumor spheroids grown in 2D and 3D. **a** Expression of EMT and CSC markers in HNSCC cells grown in 2D and cells grown in 3D. Cells cultured for 1, 3 and 7 day in 3D conditions were subjected for analysis. **b** Expression of EGFR in HNSCC tumor cells and corresponding 3D cultures collected after 1, 3 and 7 days in culture. Representative blot images and the values of the relative signals obtained by probing with selected antibody normalized to GAPDH are shown

In order to further characterize HNSCC-derived spheroids we evaluated the influence of the spherical culture conditions on EMT and CSC profile of spheroid-forming tumor cells. The expression of six EMT-associated genes (CDH1, CDH2, VIM, FN1, TWIST, FOXC2) and three stem cell markers (CD44, SOX2 and NANOG) were analyzed by RT-qPCR (Table 2) and Western blot (Fig. 3a). In contrast to the corresponding 2D monolayer, a higher expression of CDH1 mRNA in the 3D cultures after 7 days of cultivation was observed. The EMT phenotype of the analyzed HNSCC spheroids was represented by upregulation of single EMT-related genes and no evident pattern could be depicted. However, the most epithelial phenotype was preserved in LK1108 spheroids (all analyzed EMT-associated genes down regulated), which also were the steadiest spheroids as compared to LK0858B, LK0902 and LK1122 spheroids (Table 2). All analyzed cell lines showed an increased expression of the CSC-associated transcription factors such as NANOG and SOX2 in 3D cultures in comparison to 2D monolayers. In two of the cell lines, LK1108 and LK0902, the qPCR results were also confirmed at the protein level (Fig. 3a). Moreover, the protein expression of EGFR after 3 and 7 days of 3D culturing was decreased in three cell lines (LK0858B, LK0917 and LK1122) and in the other two cell lines (LK0902 and LK1108) a clear increase of EGFR was detected (Fig. 3b).

**Table 2:** mRNA expression of EMT and CSC markers in 7 days old HNSCC tumor spheroids relative to cells cultured in 2D

Gene	LK1108	LK0917	LK0858B	LK1122	LK0902
NANOG	*11.1*	*1.9*	*4.7*	*26.8*	*11.3*
SOX2	*11.7*	*1.4*	*13.3*	*22.9*	*20.8*
CD44	1.0	1.1	*1.5*	1.0	***0.6***
CDH1	*1.5*	*1.4*	*2.8*	*1.6*	*8.8*
CDH2	***0.1***	***0.6***	***0.1***	*5.8*	***0.1***
FN1	***0.1***	***0.8***	***0.2***	1.0	*2.0*
FOXC2	***0.1***	***0.8***	*1.5*	*3.9*	***0.1***
TWIST	***0.8***	*1.4*	*5.9*	0.9	*2.1*
VIM	***0.1***	***0.4***	***0.1***	1.0	***0.2***

Upregulated genes—italics, downregulated genes—bold italics; each value represents the mean of two independent experiments

### Treatment response in 3D HNSCC spheroids reveals resistance induction

**Fig. 4:**
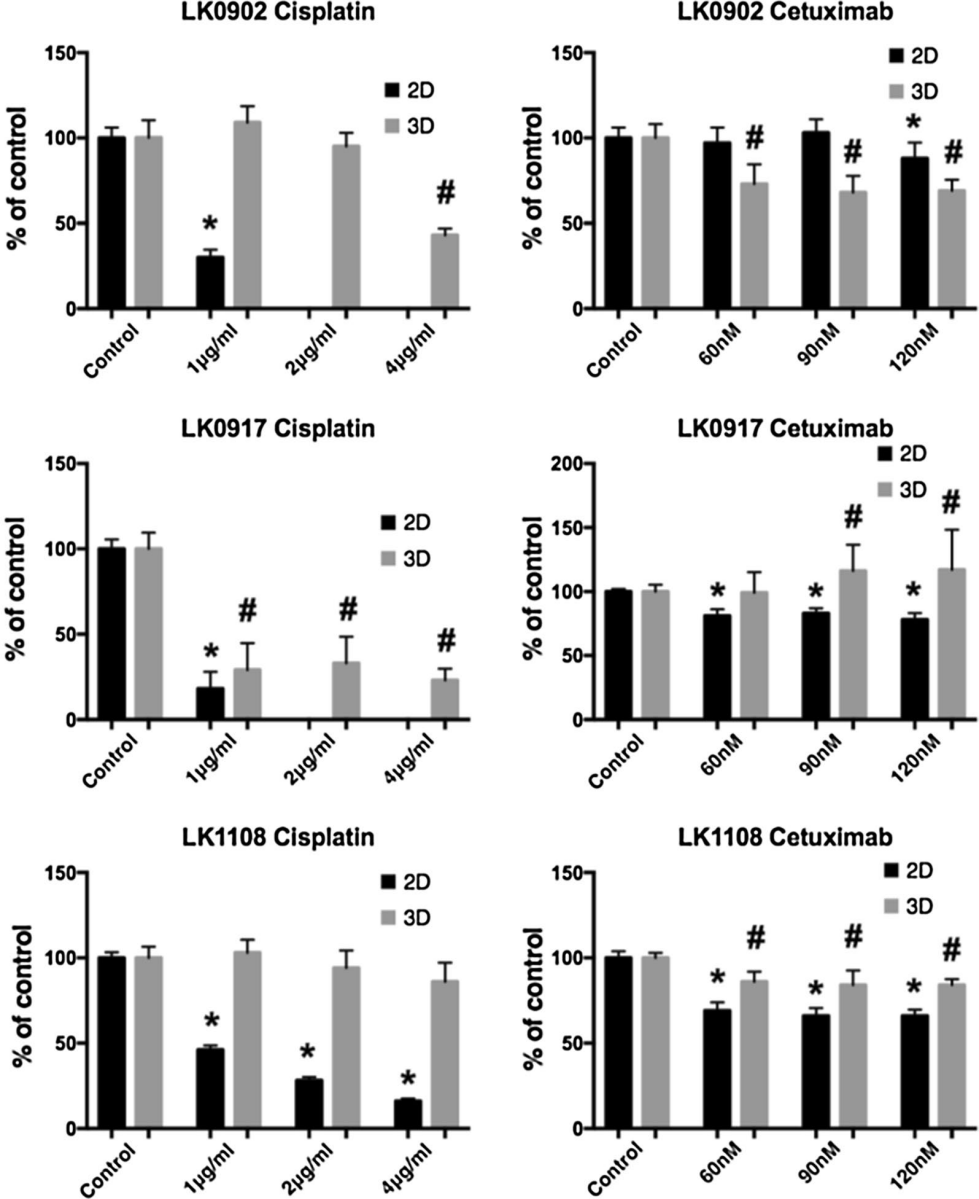
Cell viability of HNSCC cells grown in 2D and 3D after treatment with cisplatin and cetuximab. The cell viability upon treatment with cisplatin and cetuximab was measured by the MTS assay. Absorbance was measured at λ = 490 nm using an ELISA microplate reader. All measurements were carried out in triplicates and the data are shown as a mean of ± SD. The data are shown as a mean of ± SD; *p < 0.05 (2D) and ^#^p < 0.05 (3D) according to Student’s test

**Fig. 5:**
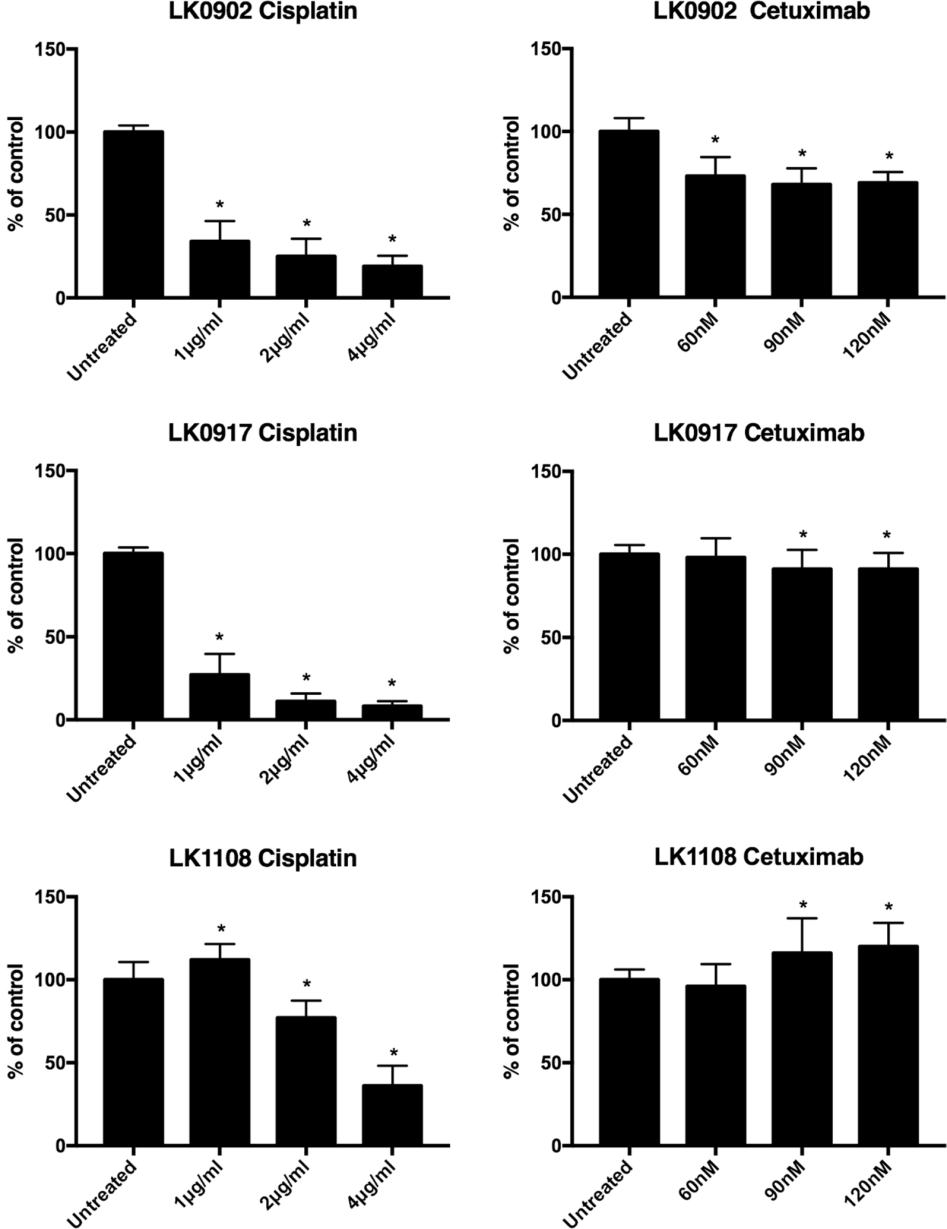
In vitro clonogenic assay in HNSCC spheroid-derived cells after treatment with cisplatin and cetuximab. Crystal violet staining was used to assess longstanding effect of cisplatin and cetuximab on the capability of HNSCC spheroid-derived cells to form colonies. Cell proliferation is presented as the percentage of the untreated control. All measurements were carried out in triplicates and the data are shown as a mean of ± SD; *p < 0.05

**Fig. 6:**
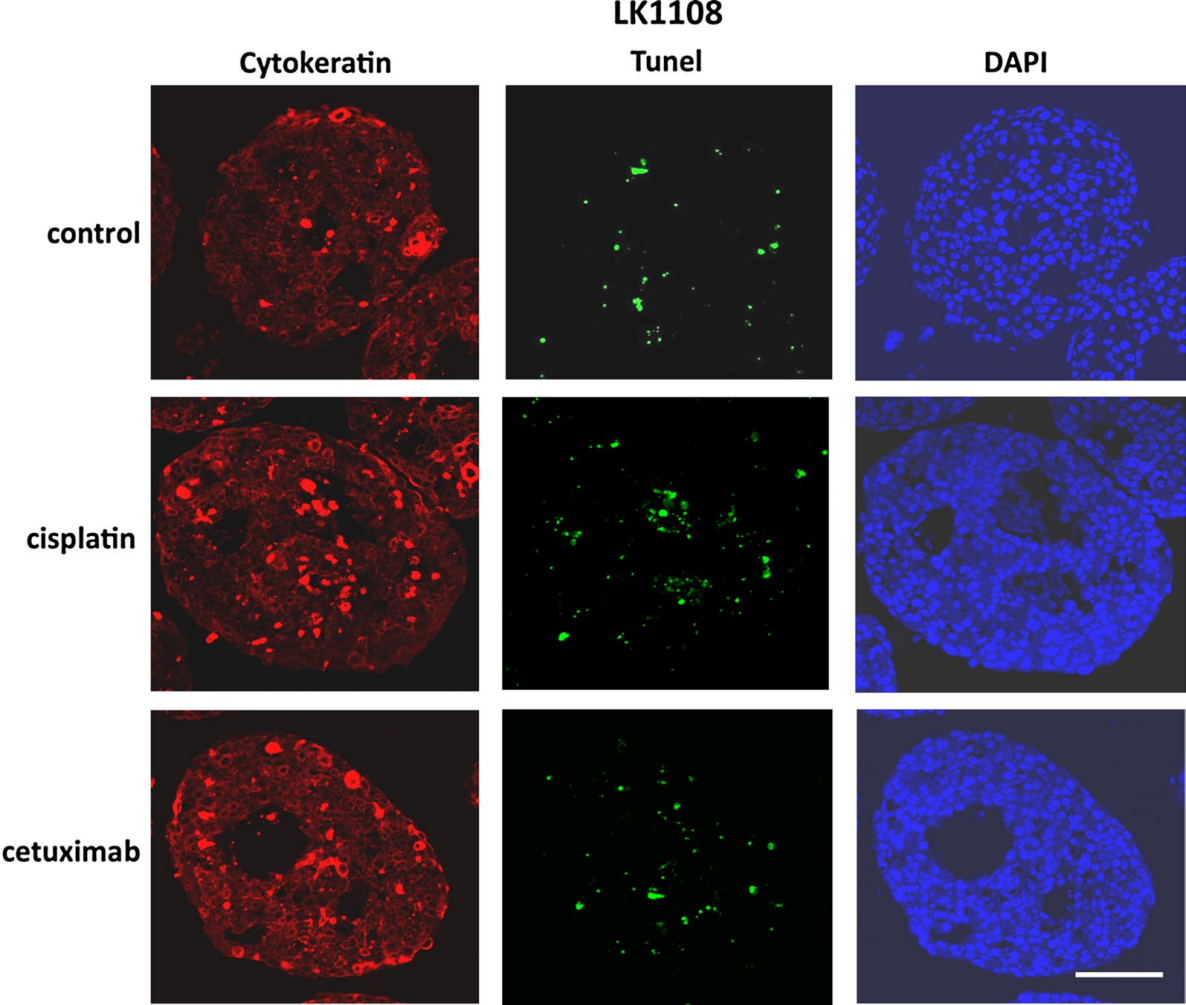
TUNEL staining of HNSCC tumor spheroids. Cancer cells within the spheroids are stained with cytokeratin (red). TUNEL positive apoptotic cells (green) are detected at 7 days after treatment with cisplatin or cetuximab. Nuclei are counterstained with DAPI (blue); scale bar = 100 µm

In three cell lines with different expression of EGFR and EMT markers changes in treatment response to cisplatin (1, 2 and 4 µg/ml) and cetuximab (60, 90 and 120 nM) were investigated in cells cultured in 3D compared to 2D monolayers using a MTS-based assay (Fig. 4). Overall, cells grown in 3D tumor spheroids showed higher viability after treatment with increasing doses of cisplatin and cetuximab. Interestingly, the LK0902 cells were relatively more sensitive to cetuximab treatment in 3D conditions than cells grown in 2D. Next, we investigated the capacity of spheroid-derived cells to proliferate after treatment with cisplatin and cetuximab by in vitro clonogenic assay. As shown in Fig. 5, all analyzed spheroid-derived cells exhibit cellular cisplatin sensitivity with the lowest surviving fraction for the same drug dose in LK0917 cell line. In comparison to cisplatin, reduced viability of spheroid-derived cells after cetuximab treatment was observed only in LK0902 cell line. The cetuximab resistance in our 3D model was evident in the LK0917 and LK1108 cell lines. Additionally, induction of apoptotic cell death by cisplatin and cetuximab in the analyzed tumor spheroids was confirmed by TUNEL staining (Fig. 6).

## Discussion

Our long-term goal is to generate an in vitro model where HNSCC cells and cancer-associated fibroblasts (CAFs) from the same patient are cultured together in 3D and can be used in the search for new predictive markers and new targets for anti-cancer treatment. Moreover, the 3D tumor spheroids mimic the in vivo tumor morphology in comparison to conventional 2D cultures. The advantages of using 3D model for in vitro studies in cancer research have been reported in the last years [6, 17, 18]. The differences between 2D and 3D models should be assessed in order to understand the in vitro complexity of the tumor. Furthermore, we need to establish reliable methods for measurement of cell death, cell viability and cell proliferation for cells growing in spheroids.

Most studies using 3D models have been performed with cancer cell lines originating from earlier decades and numerous passages of cells in vitro is known to cause genetic alterations and different behaviour when exposed to various treatments. Although some 3D HNSCC cell culture models have been published, they mostly use high passages of commercially available cell lines such as FaDu, SCC25 or CAL27 HNSCC cell lines [19, 20]. In our study, we use recently established cell lines from our own collection of tumor-derived cell lines (from tumors in the tongue, larynx and gingiva) that reflect the actual tumor behaviour better. When these cell lines were cultured in 3D, the expression of CDH1 and the expression of the stem cells markers Nanog and Sox2 were increased compared to 2D (Table 2). It has recently been published that the morphology of HNSCC spheroids was related to E-cadherin and Ki67 expression [20]. The upregulation of E-cadherin seems to be important for the formation of tumor spheroids along with decrease in DNA synthesis as visualized by Ki67 staining.

The morphology of the spheroids differed considerably between cell lines after 7 days of culture and a correlation between down regulation of EMT-associated proteins and stable growth in spheroids was observed (Fig. 1). LK1108 cells formed the most stable spheroids and all investigated EMT proteins were downregulated in 3D compared to 2D culture. Others have also shown that cell lines exhibiting an epithelial phenotype produce more regular and even aggregates [21]. In contrast, cells from LK0858, LK0902 and LK1122 spheroids started to migrate to the medium after about 3 days of culture, probably due to the up regulation in some of the investigated EMT-associated genes when cultured in 3D (Table 2). This migration of cancer cells into the medium can be linked to the invasive capacity of these cell lines.

One aim of this study was to compare two cellular systems in terms of viability of HNSCC cells after treatment with cisplatin and cetuximab. All analyzed cell lines (LK0902, LK0917 and LK1108) cultured in 3D were less sensitive to cisplatin compared to cells cultured in 2D (Fig. 4). We have previously showed that HNSCC cell populations with increased expression of Nanog and Sox1 possess an EMT phenotype and are more resistant to cisplatin treatment [22]. Possibly, the increased resistance to cisplatin treatment is due to the increased expression of these CSC-associated proteins (Nanog and Sox1) observed in the tumor spheroids. In two of the analyzed cell lines, LK0917 and LK1108, a 3D culture-mediated resistance to cetuximab was observed (Fig. 4). In LK0917 cell line, an increased proliferation was detected after cetuximab treatment (Fig. 5), which was probably due to its very low basal expression of EGFR in untreated spheroids (Fig. 3c). We have previously shown that the LK0855 HNSCC cell line with a lower expression of EGFR than normal oral keratinocytes, shows an increased proliferation after exposure to cetuximab compared to untreated controls [23]. Similarly, in the 3D colorectal cancer (CRC) cell lines that were cultured in laminin-rich-extracellular matrix conditions, the EGFR expression was decreased compared to 2D cultures. Furthermore, cells from the *KRAS* and *BRAF* wild type cell line CACO-2 show an increased resistance to the EGFR tyrosine kinase inhibitor AG1478 when cultured in 3D [24]. In the other two cell lines, LK0902 and LK1108, an increased EGFR expression was found in spheroids cultured for 3 and 7 days, and LK0902 cells showed an increased sensitivity to cetuximab when cultured in 3D (Fig. 4). In another study involving CRC cell lines, the EGFR-expressing cystic (CC) 3D cultures were more sensitive to cetuximab than the EGFR-expressing spiky (SC) 3D cultures derived from HCA-7 cell line. This was due to decreased tyrosine phosphorylation of MET and RON in CC cultures compared to SC cultures [25]. Moreover, EGFR and c-MET have been identified as targets of tumor-suppressive miR-1 and miR-206 in HNSCC [26].

Despite the fact that EMT has been linked to drug resistance in HNSCC [10, 27, 28], our results showed no specific pattern of EMT and drug response in the analyzed tumor spheroids pointing at other co-factors involved in drug resistance. However, our results indicate that increased expression of EMT-associated proteins increases the migration of tumor cells growing in spheroids.

## Conclusions

Taken together, we highlight advantages of using 3D culture models over traditional 2D monolayers cultures. We found that cells cultured in 3D take on the CSC-like phenotype and our results obtained from 3D culture of HNSCC cells differ significantly from 2D model in terms of drug efficacy. Interestingly, notable differences were found between the cell lines regarding changes in EGFR and EMT-associated protein expression as well as in treatment response to both cisplatin and cetuximab after 3D culturing. We believe that our model will successfully bridge the gap between 2D cultures and in vivo conditions and increases the chance for reliable predictive markers in HNSCC.

## Additional file

**Additional file 1: Figure S1.** Histological evaluation of HNSCC-derived tumor spheroids. Representative fluorescent microscopy images of TUNEL assay for identification of apoptotic cells (*green*) in HNSCC tumor spheroids along with cytokeratin staining (*red*) for identification of tumor cells within the spheroids. Nuclei are counterstained with DAPI (*blue*); scale bar = 50 µm.

## Abbreviations

HNSCC: Head and Neck Squamous Cell Carcinoma
EMT: epithelial–mesenchymal transition
CSCs: cancer stem cells
IF: immunofluorescence
EGFR: epidermal growth factor receptor
CAFs: cancer-associated fibroblasts
CRC: colorectal cancer
