# Supplementary figures and images for: The effect of 2D and 3D cell cultures on treatment response, EMT profile and stem cell features in head and neck cancer
Source: Cancer Cell Int. 2019 Jan 14;19:16. doi: 10.1186/s12935-019-0733-1 (PMC6332598; doi:10.1186/s12935-019-0733-1)

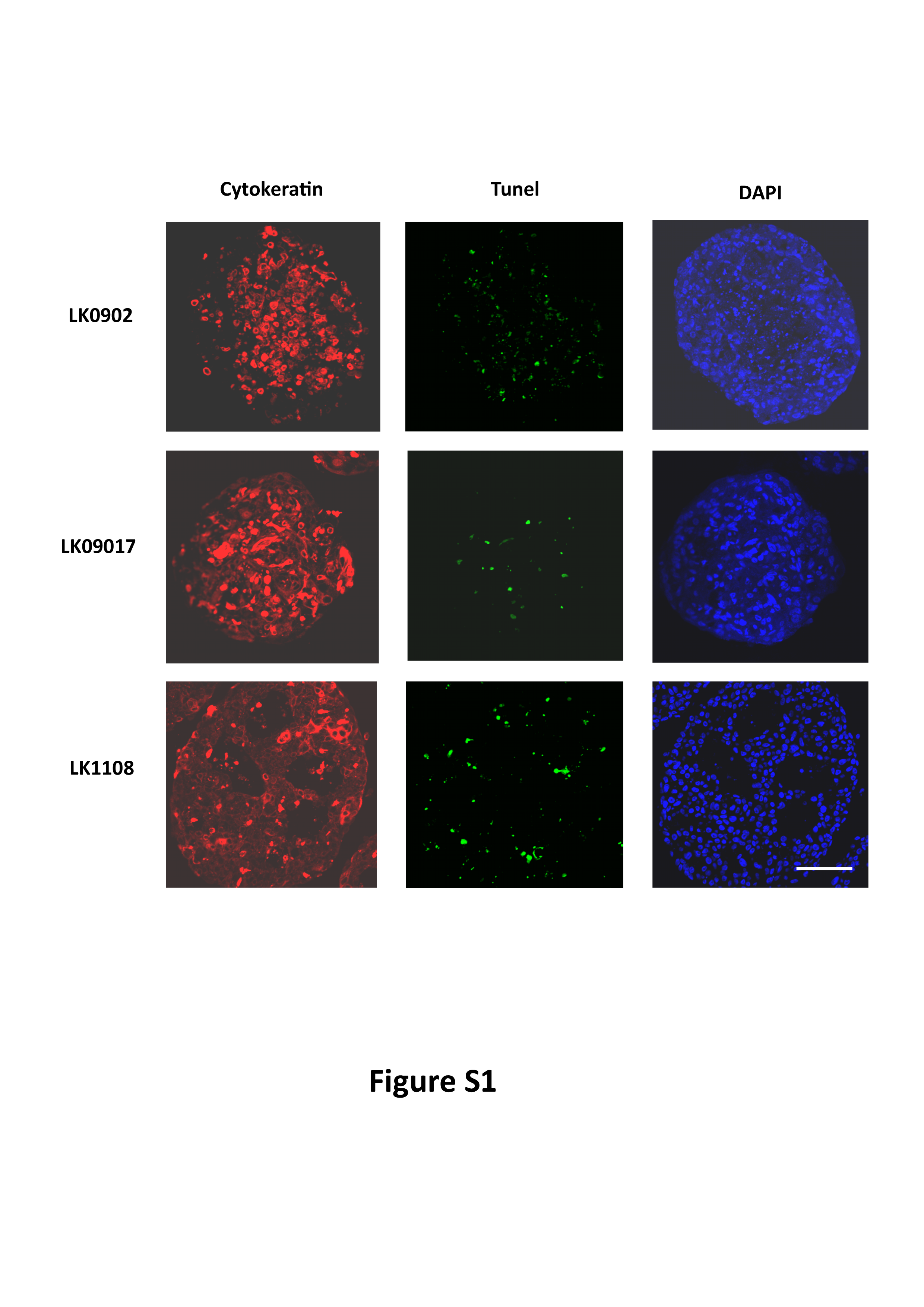

Supplement: Supplementary file 1 — Additional file 1: Figure S1. Histological evaluation of HNSCC-derived tumor spheroids. Representative fluorescent microscopy images of TUNEL assay for identification of apoptotic cells (green) in HNSCC tumor spheroids along with cytokeratin staining (red) for identification of tumor cells within the spheroids. Nuclei are counterstained with DAPI (blue); scale bar = 50 µm. [file 12935_2019_733_MOESM1_ESM.tif]
